# Mobile App–Induced Mental Fatigue Affects Strength Asymmetry and Neuromuscular Performance Across Upper and Lower Limbs

**DOI:** 10.3390/s25154758

**Published:** 2025-08-01

**Authors:** Andreas Stafylidis, Walter Staiano, Athanasios Mandroukas, Yiannis Michailidis, Lluis Raimon Salazar Bonet, Marco Romagnoli, Thomas I. Metaxas

**Affiliations:** 1 Laboratory of Evaluation of Human Biological Performance, Department of Physical Education and Sports Sciences, Aristotle University of Thessaloniki, University Campus of Thermi, 57001 Thessaloniki, Greece; amandrou@phed.auth.gr (A.M.); ioannimd@phed.auth.gr (Y.M.); tommet@phed.auth.gr (T.I.M.); 2 Department of Physical Education and Sport, University of Valencia, 46000 Valencia, Spain; walterstaiano@gmail.com (W.S.); marco.romagnoli@uv.es (M.R.); 3 International University SEK, Quito 170151, Ecuador; raimon.salazar@uisek.edu.ec

**Keywords:** mental fatigue, psychomotor vigilance, isometric strength, handgrip asymmetry, Stroop task, squat jump, perceived exertion, cognitive load, sport performance, neuromuscular fatigue

## Abstract

This study aimed to investigate the effects of mental fatigue on physical and cognitive performance (lower-limb power, isometric and handgrip strength, and psychomotor vigilance). Twenty-two physically active young adults (12 males, 10 females; M_age_ = 20.82 ± 1.47) were randomly assigned to either a Mental Fatigue (MF) or Control group (CON). The MF group showed a statistically significant (*p* = 0.019) reduction in non-dominant handgrip strength, declining by approximately 2.3 kg (about 5%), while no such change was observed in the CON group or in dominant handgrip strength across groups. Reaction time (RT) was significantly impaired following the mental fatigue protocol: RT increased by 117.82 ms, representing an approximate 46% longer response time in the MF group (*p* < 0.001), whereas the CON group showed a smaller, non-significant increase of 32.82 ms (~12% longer). No significant differences were found in squat jump performance, indicating that lower-limb explosive power may be less affected by acute mental fatigue. These findings demonstrate that mental fatigue selectively impairs fine motor strength and cognitive processing speed, particularly reaction time, while gross motor power remains resilient. Understanding these effects is critical for optimizing performance in contexts requiring fine motor control and sustained attention under cognitive load.

## 1. Introduction

Mental fatigue (MF) is a psychobiological state arising from prolonged cognitive effort, typified by heightened subjective fatigue, diminished cognitive functioning, and reduced motivational drive to persist in the task at hand [1,2,3]. In the realm of sport, MF is particularly pertinent, as athletes frequently encounter cognitively taxing situations such as tactical analysis, travel-related fatigue, media duties, and real-time decision-making during competition [4,5,6]. MF has been shown to impair both sport-specific psychomotor responses—such as reaction time and movement accuracy [7]—and physical capacities, including running performance [8,9].

Despite increasing interest, findings regarding MF’s impact on physical performance remain inconsistent across exercise modalities and task characteristics [3]. In a review of 29 studies, Pageaux and Lepers [3] noted that while MF does not impair maximal force output, it significantly hinders endurance, motor skill execution, and decision-making—largely through an elevated perception of effort. These results are echoed in Staiano et al. [10], who observed that participants experiencing MF perceived resistance loads as 7–15% heavier, despite stable strength output. Mechanistically, such decrements have been linked to a psychobiological model involving altered cost–benefit valuation, reduced dopaminergic tone in the anterior cingulate cortex, and suppressed motor pathway excitability [11,12].

With respect to maximal strength (e.g., one-repetition maximum [1RM] efforts), the existing literature indicates that MF does not significantly affect bench press or squat 1RM, even when induced through cognitively demanding tasks such as the Stroop or social media use [10,13]. Neurophysiological assessments have corroborated these findings, revealing unaltered corticospinal excitability and neuromuscular recruitment patterns during maximal voluntary contractions [14,15]. In contrast, submaximal strength endurance is markedly impaired. Alix-Fages et al. [16] reported an approximate 40% reduction in repetitions to failure at 65–80% 1RM following MF induction. These impairments are not mirrored by physiological fatigue markers such as blood lactate or heart rate but are instead associated with disproportionate increases in perceived exertion (RPE) and effort miscalibration [10,17], underscoring the psychological nature of MF’s impact on task persistence.

Concerning handgrip strength, Jacquet et al. [12] demonstrated that MF disrupts hand force output through cortical-level alterations, including attenuated attention-related ERP components and elevated alpha-band inhibitory activity—factors which diminish motor drive and coordination without directly altering muscle activation or motivation. Notably, grip strength in the non-dominant hand is more adversely affected by MF than in the dominant hand [12,18], likely due to the increased cortical resources needed to control the less-automated limb [18,19]. Movements involving the non-dominant side or unilateral movements are generally more attentionally demanding and less efficient, rendering them more susceptible to central interference and executive resource depletion [19,20]. 

Conversely, single maximal efforts—such as the countermovement jump (CMJ)—appear largely resistant to MF effects [13,17]. However, when such tasks are repeated over time, performance declines become evident. Specifically, repeated jump ability (RJA) tests show reduced mean jump height and elevated fatigue index under MF [21]. Crucially, these changes are not paralleled by physiological signs of fatigue, such as lactate accumulation or heart rate elevation, supporting the central origin of the impairment [21,22]. Additional markers, such as heightened RPE and psychomotor vigilance deterioration (+16–18%) highlight altered motor planning and attentional fluctuations that compromise output consistency in repeated high-intensity movements [21,23].

While numerous studies confirm MF-related impairments in submaximal tasks, some findings suggest a more nuanced picture. Holgado et al. [14] found that individualized cognitive load protocols did not impair neuromuscular performance in recreationally trained individuals. Their preregistered, within-subject randomized design included a high-effort mental task followed by a time-to-exhaustion cycling protocol at 80% peak power. Despite significantly elevated subjective MF ratings, neither endurance performance, perceived exertion, nor corticospinal excitability were negatively affected [14]. Similarly, Angius et al. [23] observed that MF did not compromise repeated sprint ability (RSA), although it interacted cumulatively with physical fatigue to impair psychomotor vigilance.

Building on these findings, the present study aims to clarify whether previously observed null effects of MF generalize across additional physical domains—specifically lower-limb power (squat jump), isometric strength (back and handgrip, dominant vs. non-dominant), and psychomotor vigilance—in a sample of physically active young adults with varied athletic backgrounds. Given earlier evidence of limb-specific vulnerability and distorted effort perception under MF, we adopt a targeted approach to assess whether asymmetrical strength responses and motor-cognitive coupling moderate fatigue effects.

We hypothesized that mental fatigue would selectively impair non-dominant handgrip strength and psychomotor vigilance performance, while leaving lower-limb power and dominant-limb strength largely unaffected.

## 2. Materials and Methods

### 2.1. Participants

The total sample consisted of 22 participants (N = 22), with a mean age of 20.82 years (SD = 1.468), ranging from 20 to 24 years. The sample was relatively balanced in terms of gender, comprising 12 males (54.5%) and 10 females (45.5%). Participants had a mean height of 173.55 cm (SD = 10.01, range = 155–191 cm) and a mean weight of 68.48 kg (SD = 12.14, range = 52–93.5 kg). They were randomly assigned to one of two experimental groups using gender-stratified randomization in Microsoft Excel, ensuring gender balance: Mental Fatigue (MF) or Control (CON), with 11 individuals in each. In the MF condition (*n* = 11), the mean age was 20.27 years (SD = 1.19), height 173.73 cm (SD = 9.84), and weight 67.07 kg (SD = 11.79), including 6 males and 5 females. In the CON group (*n* = 11), mean age was 21.36 years (SD = 1.567), height 173.36 cm (SD = 10.65), and weight 69.88 kg (SD = 12.90). Inclusion criteria required participants to be actively engaged in at least one sport and to train more than three times per week, with no musculoskeletal injuries in the previous six months. Exclusion criteria included recent illness, injury, infection, or medication use. All participants provided written informed consent. The study was approved by the Research Ethics Committee of the School of Physical Education and Sport Science at Thessaloniki (Protocol No. 203/2024).

### 2.2. Research Tools

#### 2.2.1. Soma NPT Application, Stroop Task, and Psychomotor Vigilance Task (PVT)

Cognitive performance and mental fatigue were assessed using the Soma Neuro Performance Training (Soma NPT, Lucerne, Switzerland) application—a mobile platform for iOS devices that delivers cognitive training and assessment tasks with high precision and real-time feedback. It is optimized for accurate visual stimulus delivery and touch-based interaction and includes performance metrics such as reaction time (ms), accuracy, response consistency (coefficient of variation), and Rate Correct Score (RCS). Soma NPT includes multiple configurable cognitive tasks, such as the Stroop task, which can be adjusted for difficulty, timing, feedback modality, and execution modes. The app has been validated in numerous peer-reviewed studies, on mental fatigue and brain endurance training (BET) [10,21,24,25,26,27,28,29,30,31,32,33,34]. In the present study, a 20 min Stroop task was used to induce mental fatigue. The Stroop task [35], based on the classic Stroop effect, challenges participants to name the ink color of color-denoting words (e.g., “BLUE” in red ink), requiring attentional control and cognitive inhibition. Protocols lasting more than 15 min have been shown to reliably elicit mental fatigue [36,37,38,39]. However, the present stroop was completed in an adaptive mode as mentioned by Staiano et al. (2024) [32]. This mode adapts the difficulty based on participant response and it proved to induce MF faster compared to a classic Stroop.

The Psychomotor Vigilance Task (PVT) [40] was also administered using the SOMA-NPT app. This task measures sustained attention and is sensitive to mental fatigue effects. Participants responded to randomly appearing visual stimuli over 5 min at three time points: pre-condition (baseline), post-condition (after MF or CON), and following physical performance tests. Key metrics included mean reaction time and the number of lapses (responses > 500 ms), in line with prior MF research [10,27].

#### 2.2.2. Mental Fatigue Visual Analog Scale (M-VAS)

The M-VAS is a psychometric tool for assessing perceived mental fatigue and has been applied across diverse experimental settings, including resistance training [10], dynamic calisthenics [24], and soccer-specific technical tasks in team sport contexts [29]. It has also shown sensitivity to age-related changes in fatigue perception [27], to variations in task characteristics [41], and the effectiveness of countermeasure interventions targeting mental fatigue [42]. Participants marked their fatigue on a 10 cm line anchored with “not at all fatigued” and “completely fatigued.” Fatigue was described as a subjective state of cognitive exhaustion and reduced attentional capacity [5]. The scale was used before and after each condition to quantify changes in self-reported fatigue.

#### 2.2.3. NASA Task Load Index (NASA-TLX)

The NASA-TLX [43] measures perceived workload across six dimensions: mental, physical, and temporal demand; performance; effort; and frustration. Administered post-condition, it served to assess cognitive load during the tasks. It is frequently used in MF research to confirm task-induced cognitive strain [32,44,45].

#### 2.2.4. Rating of Perceived Exertion (RPE)

Immediately after each task, participants rated their exertion using Borg’s 0–10 scale [46].

#### 2.2.5. My Jump Lab Application (My Jump 3)

Jump performance was assessed using My Jump 3, a validated smartphone app that calculates vertical jump height from high-speed video [47]. Participants performed three maximal squat jumps (SJ), recorded using an iPhone 15 Pro at 240 FPS, with the device positioned following validated protocols [48,49]. Jump height was calculated from flight time using AI-driven frame detection. This approach aligns with standard field-based methods [50,51].

#### 2.2.6. Takei 5401 Hand Grip Digital Dynamometer

Grip strength was measured using the Takei T.K.K. 5401 dynamometer (Takei Scientific Instruments, Niigata, Japan), which has high accuracy (±2.0 kgf) and excellent reliability (r > 0.90) [52,53]. Participants stood upright with arms at their sides and wrists neutral. Each hand was tested twice, starting with the dominant side, with 60 s rest between trials [52,54]. The best value (kg) was used for analysis.

#### 2.2.7. Takei 5402 Back Muscle Dynamometer

Back strength was assessed using the T.K.K. 5402 dynamometer (Takei, Niigata, Japan), which records force from isometric pulling with validated accuracy (±6 kgf) [55,56,57]. Participants stood with a lumbar flexion of ~30°, pulled the handle upward without bending the knees, and performed two trials with 1 min rest. The higher value was recorded [58].

### 2.3. Research Procedure

**Figure 1:**
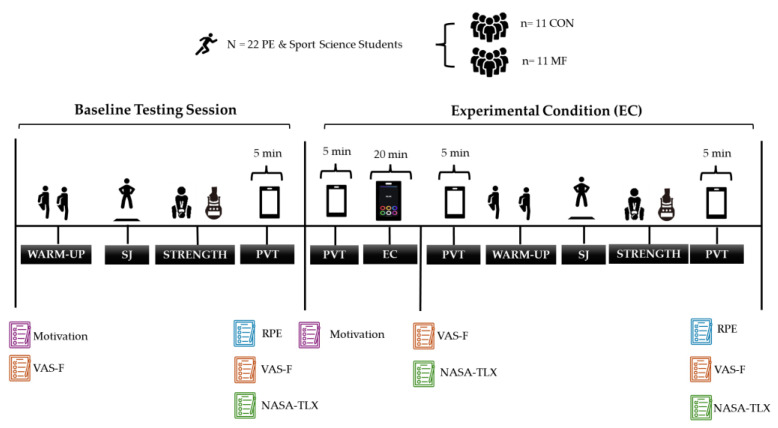
Overview of the experimental protocol. Note: The figure illustrates the sequence of procedures for both groups, including warm-up, squat jump (SJ), isometric back strength (STRENGTH), psychomotor vigilance test (PVT), and exposure to the experimental condition (EC: Stroop task for the Mental Fatigue group [MF] or a control activity for the Control group [CON]). PVT = Psychomotor Vigilance Test; SJ = Squat Jump; EC = Experimental Condition; MF = Mental Fatigue group; CON = Control group. Baseline indicates pre-intervention; EC indicates post-intervention.

This randomized between-subjects study involved three visits: familiarization, baseline testing, and experimental condition (EC). Participants were randomly assigned to MF or CON conditions (Figure 1). Familiarization included procedural orientation. Pre-test instructions matched prior MF studies [21], including 7+ h of sleep and abstention from stimulants or exercise. Laboratory conditions and testing time were standardized. Baseline testing began with self-reports of M-VAS and motivation [21], followed by a dynamic warm-up. Participants completed squat jumps (SJ) [50,51], back strength testing using the Takei 5402 [55,56,57], and handgrip testing [52,54], then performed a 5 min PVT. Finally, they completed M-VAS, RPE, and NASA-TLX scales. A week later, the EC session began with filling the motivation questionnaire and with a 5 min PVT. The MF group then performed a 20 min Stroop task (SOMA-NPT), while the CON group viewed a neutral documentary with minimal cognitive demand [59]. After the condition, participants repeated the PVT, self-ratings (M-VAS and NASA TLX), warm-up, and all physical tests in the same order and conditions as baseline. A final PVT and all self-report measures (RPE, M-VAS, NASA TLX) were completed at the end.

### 2.4. Statistical Analysis

An a priori power analysis was conducted using G*Power (Version 3.1.9.7) to determine the minimum required sample size for detecting a statistically significant Group × Time interaction in a 2 (Group: Experimental vs. Control) × 2 (Time: Pre vs. Post) mixed-design ANOVA. The analysis employed the “Repeated Measures: Within–Between Interaction” module, with the following assumptions: a large effect size (f = 0.40), an alpha level of 0.05, statistical power of 0.80 (1–β), and an assumed correlation of 0.50 between repeated measures. The nonsphericity correction (ε) was set to 1, as the assumption of sphericity is not violated when only two time points are analyzed. This calculation indicated a minimum total sample size of 16 participants (8 per group). To enhance power and account for potential dropouts or variability, 11 participants per group (N = 22) were recruited. This decision was based not only on the statistical requirements but also on methodological precedent. Specifically, Queirós et al. [60] conducted a similar study on the effects of mental fatigue using a within-subjects cross-over design with nine participants who completed all procedures. Their sample size estimation was based on a large effect size (ES = 1.05) derived from a small pilot sample. In contrast, the present study employed a between-subjects design, which typically entails greater inter-individual variability than within-subject approaches. Therefore, a more conservative effect size assumption (ES = 0.40) was adopted in our power analysis, and two additional participants per group were included compared to Queirós et al. [60], thereby enhancing statistical power and compensating for the absence of within-subject control. Descriptive statistics (M ± SD unless otherwise stated) were calculated for all outcome variables. After testing for normality using Shapiro–Wilk test, histograms, Q–Q plots, and boxplots, between-group differences in subjective responses were analyzed. Independent samples *t*-tests were used to compare between-group differences in M-VAS and NASA-TLX ratings at specific time points following the experimental manipulation (e.g., after the cognitive task). To assess the effects of the experimental condition on physical performance variables (handgrip strength, back strength, squat jump), cognitive performance variables (PVT reaction time, PVT lapses), RPE, motivation, and NASA-TLX subscales across testing sessions, two-way mixed-design repeated-measures ANOVAs were conducted for each outcome, with Time (Pre vs. Post) as the within-subjects factor and Group (Mental Fatigue vs. Control) as the between-subjects factor. Where significant interactions were found, follow-up analyses included Bonferroni-adjusted post hoc comparisons and Cohen’s d was calculated for each significant pairwise contrast. Mauchly’s test of sphericity was applied where appropriate; Greenhouse–Geisser corrections were used if assumptions were violated. Effect sizes were reported using partial eta squared (η^2^ₚ) and interpreted as follows: small (0.01 ≤ η^2^ₚ < 0.06), medium (0.06 ≤ η^2^ₚ < 0.14), and large (η^2^ₚ ≥ 0.14), based on conventions proposed by Cohen [61] and Richardson [62]. For *t*-tests, Cohen’s d [61] was interpreted as small (d < 0.50), medium (d ≥ 0.50 and < 0.80), and large (d ≥ 0.80). Specifically, for Cohen’s d, 95% confidence intervals were calculated, as the metric can take both positive and negative values and is associated with symmetric two-tailed tests. In contrast, 90% confidence intervals were reported for partial eta squared (η^2^ₚ), in accordance with methodological recommendations suggesting that a 90% CI is more appropriate given the one-sided nature of F-tests used in ANOVA analyses [63,64]. This approach aimed to avoid interpretative inconsistencies, as 95% CIs for η^2^ₚ may include zero even when *p* < 0.05, due to the inherently positive and bounded nature of squared effect size indices [65]. Confidence intervals for η^2^ₚ were computed using SPSS syntax developed by Wuensch [64], based on method described by Smithson [66]. All analyses were performed using Jamovi (Version 2.3.28) and IBM SPSS Statistics (Version 29.0.2.0). The alpha level was set at *p* < 0.05 for all tests.

## 3. Results

**Figure 2:**
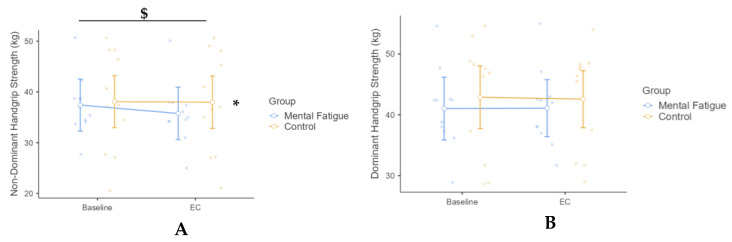
Effect of Mental Fatigue on Non-Dominant (**A**) and Dominant Handgrip Strength (**B**). Note: Handgrip strength (mean ± SE) for the Mental Fatigue (MF) and Control (CON) groups across sessions (Pre vs. Post). Symbols indicate: $ = significant Group × Session interaction; * = significant main effect of Session (Time). (**A**) Non-Dominant Handgrip Strength: A significant Group × Session interaction ($ *p* < 0.05) and a significant main effect of Session (* *p* < 0.05) were observed for non-dominant handgrip strength. (**B**) Dominant Handgrip Strength: No significant effects were found for dominant handgrip strength.

Non-dominant handgrip strength was analyzed using a two-way mixed-design repeated-measures ANOVA, with Session (Pre vs. Post) as the within-subjects factor and Group (MF vs. CON) as the between-subjects factor. A significant Group × Session interaction was observed, *F*(1, 20) = 4.94, *p* = 0.038, η^2^ₚ = 0.198, 90% CI [−0.01, 0.41], reflecting a large effect. Post hoc Bonferroni-adjusted comparisons indicated that non-dominant handgrip strength significantly declined from pre- to post-test in the MF group (*p* = 0.019, Cohen’s d = 0.20, 95% CI [0.02, 0.40]), while no significant change was detected in the CON group (*p* = 0.99). A significant main effect of Session was found, *F*(1, 20) = 6.30, *p* = 0.021, η^2^ₚ = 0.240, 90% CI [−0.022, 0.451], also representing a large effect, indicating an overall reduction in strength across sessions. The main effect of Group was not significant, *F*(1, 20) = 0.174, *p* = 0.681, η^2^ₚ = 0.009, suggesting no overall difference in non-dominant handgrip strength between the MF and CON groups (Figure 2).

Dominant handgrip strength was analyzed using a two-way mixed-design repeated-measures ANOVA. The Group × Session interaction was not significant, *F*(1, 20) = 0.31, *p* = 0.586, η^2^ₚ = 0.015, indicating no differential change in dominant handgrip strength between groups over time. The main effect of Session was also not significant, *F*(1, 20) = 0.14, *p* = 0.716, η^2^ₚ = 0.007, suggesting that dominant handgrip strength remained stable across sessions. Similarly, the main effect of Group was not significant, *F*(1, 20) = 0.25, *p* = 0.620, η^2^ₚ = 0.013, indicating no overall difference in dominant handgrip strength between the MF and CON groups.

**Figure 3:**
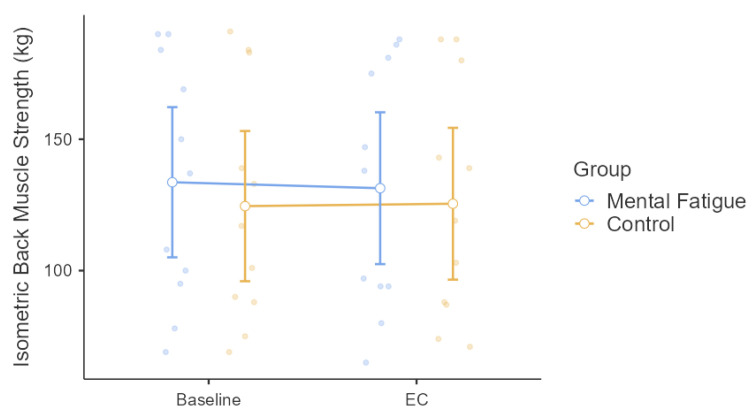
Effect of Mental Fatigue on Isometric Back Muscle Strength. Note: Isometric back strength (mean ± SE; measured in kilograms [kg]) for the Mental Fatigue (MF) and Control (CON) groups across sessions (Pre = baseline, EC = experimental condition). No significant Group × Time interaction, main effect of Time, or main effect of Group was observed. MF = Mental Fatigue group; CON = Control group; EC = Experimental Condition.

Isometric back strength was analyzed using a two-way mixed-design repeated-measures ANOVA. The Group × Time interaction was not significant, *F*(1, 20) = 2.76, *p* = 0.112, η^2^ₚ = 0.121, indicating no differential change between the Mental Fatigue and Control groups across time. The main effect of Time was also non-significant, *F*(1, 20) = 0.51, *p* = 0.484, η^2^ₚ = 0.025, showing no overall change in back strength from pre- to post-intervention. Similarly, the main effect of Group was non-significant, *F*(1, 20) = 0.15, *p* = 0.704, η^2^ₚ = 0.007, indicating comparable performance across groups (Figure 3).

**Figure 4:**
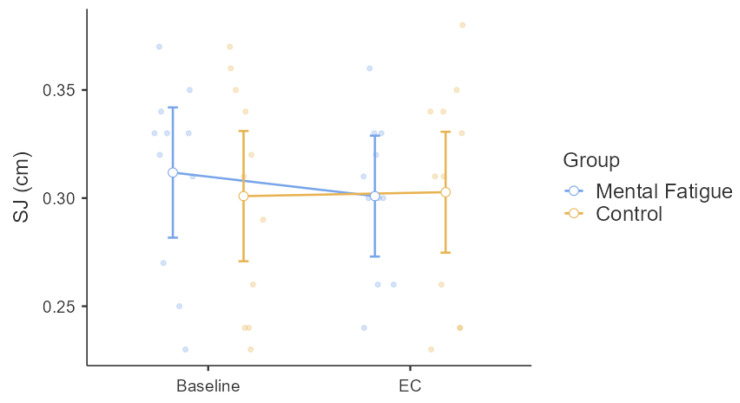
Effect of Mental Fatigue on Squat Jump (SJ). Note: Squat jump (SJ) performance (mean ± SE; measured in centimeters [cm]) for the Mental Fatigue (MF) and Control (CON) groups across sessions (Pre = baseline, EC = experimental condition). No significant Group × Session interaction, main effect of Session, or main effect of Group was observed. SJ = Squat Jump; MF = Mental Fatigue group; CON = Control group; EC = Experimental Condition.

Squat jump (SJ) height was analyzed using a two-way mixed-design repeated-measures ANOVA (Figure 4). The Group × Session interaction was not significant, *F*(1, 20) = 3.67, *p* = 0.070, η^2^ₚ = 0.155, indicating no differential change in squat jump performance between groups over time. There was no significant main effect of Session, *F*(1, 20) = 1.87, *p* = 0.186, η^2^ₚ = 0.086, showing no overall change across sessions. The main effect of Group was also non-significant, *F*(1, 20) = 0.05, *p* = 0.817, η^2^ₚ = 0.003, indicating similar squat jump performance between the MF and CON groups. While none of the main effects or interactions reached statistical significance, visual inspection of Figure 3 and Figure 4 reveals considerable inter-individual variability in both isometric back strength and squat jump performance across sessions. This variability, although not captured by the group-level statistics, may reflect individual differences in response to the experimental conditions.

**Figure 5:**
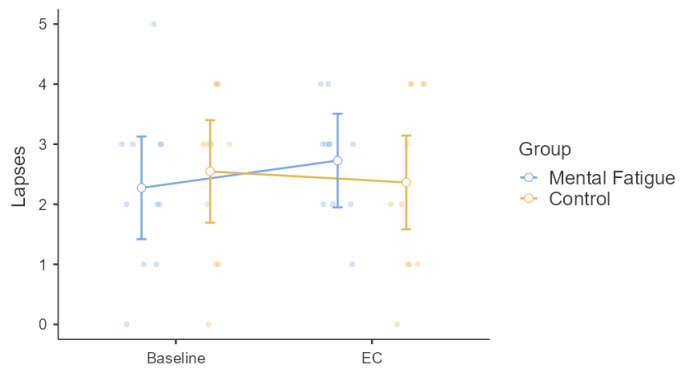
Effect on Psychomotor Vigilance (PVT) Lapses. Note: Psychomotor vigilance lapses (mean ± SE; number of lapses defined as responses > 500 ms) for the Mental Fatigue (MF) and Control (CON) groups across sessions (Pre = baseline, EC = experimental condition). No significant Group × Time interaction, main effect of Time, or main effect of Group was observed. MF = Mental Fatigue group; CON = Control group; EC = Experimental Condition.

Psychomotor vigilance lapses were analyzed using a two-way mixed-design repeated-measures ANOVA. The Group × Time interaction was not significant, *F*(1, 20) = 1.38, *p* = 0.254, η^2^ₚ = 0.064, indicating no differential change between groups over time. There was no significant main effect of Time, *F*(1, 20) = 0.25, *p* = 0.621, η^2^ₚ = 0.012, nor a significant main effect of Group, *F*(1, 20) = 0.009, *p* = 0.926, η^2^ₚ < 0.001 (Figure 5).

**Figure 6:**
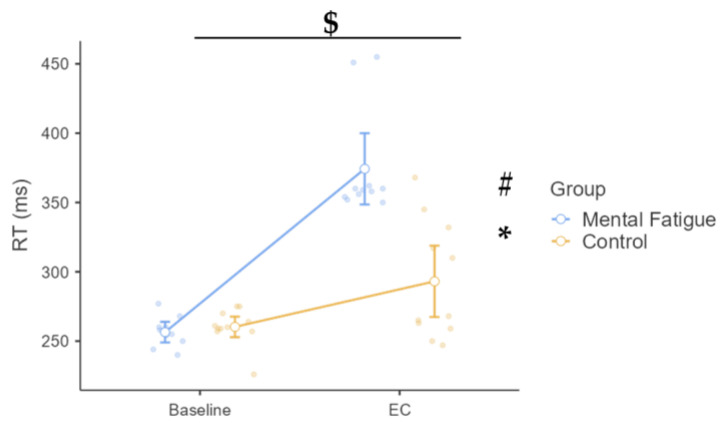
Effect on Psychomotor Vigilance (PVT) Reaction Time (RT). Note: Psychomotor vigilance reaction time (RT; mean ± SE, in milliseconds) for the Mental Fatigue (MF) and Control (CON) groups across sessions (Pre = baseline, EC = experimental condition). A significant Group × Time interaction ($ *p* < 0.05), a significant main effect of Time (* *p* < 0.05), and a significant main effect of Group (# *p* < 0.05) were observed. Symbols indicate: $ = significant condition × time interaction; * = significant main effect of time (Session); # = significant main effect of condition (Group). MF = Mental Fatigue group; CON = Control group; EC = Experimental Condition.

Psychomotor vigilance RT was analyzed using two-way mixed-design repeated-measures ANOVA. A significant Group × Time interaction was observed, *F*(1, 20) = 23.20, *p* < 0.001, η^2^ₚ = 0.537, 90% CI [0.25, 0.68], indicating a large effect. Post hoc comparisons (Bonferroni -adjusted) revealed that RT significantly increased from Pre to Post in the MF group (*p* < 0.001, Cohen’s d = −3.91, 95% CI [−6.10, −1.73]), whereas the increase in the CON group was smaller and did not reach statistical significance (*p* = 0.096). The main effect of Time was significant, *F*(1, 20) = 72.80, *p* < 0.001, η^2^ₚ = 0.785, 90% CI [0.60, 0.85], reflecting a large overall increase in RT across sessions. The main effect of Group was also significant, *F*(1, 20) = 17.20, *p* < 0.001, η^2^ₚ = 0.463, 90% CI [0.17, 0.63], with higher overall RT in the MF group compared to the CON group (Figure 6).

**Figure 7:**
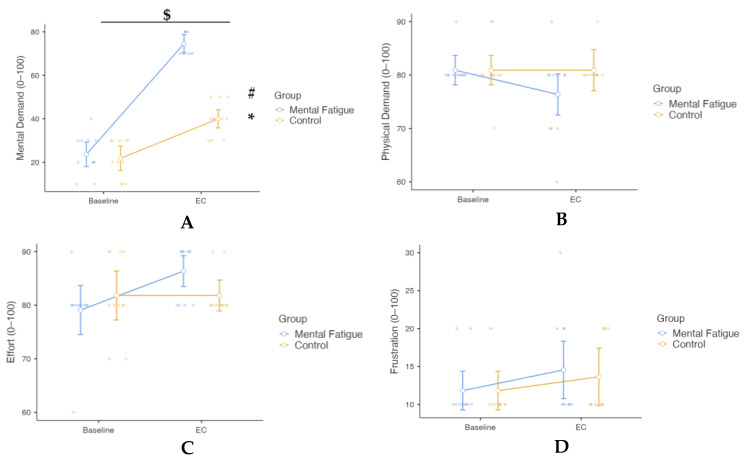
Effect of Mental Fatigue on NASA-TLX scale: Mental Demand (**A**); Physical Demand (**B**); Effort (**C**); Frustration (**D**). Note: NASA-TLX subscale ratings (mean ± SE, scale 0–100) for the Mental Fatigue (MF) and Control (CON) groups across sessions (Pre = baseline; EC = experimental condition). Symbols indicate: $ = significant Group × Session interaction; * = significant main effect of Session (time); # = significant main effect of Group (condition). MF = Mental Fatigue group; CON = Control group; EC = Experimental Condition. (**A**) Mental Demand: A significant Group × Session interaction ($ *p* < 0.05), a significant main effect of Session (* *p* < 0.05), and a significant main effect of Group (# *p* < 0.05) were observed. (**B**) Physical Demand: No significant Group × Session interaction, main effect of Session, or main effect of Group. (**C**) Effort: No significant Group × Session interaction, main effect of Session, or main effect of Group. (**D**) Frustration: No significant Group × Session interaction, main effect of Session, or main effect of Group.

Regarding the NASA-TLX (Figure 7), all subscales (mental demand, physical demand, effort, frustration) were analyzed using two-way mixed-design repeated-measures ANOVAs with Session (Pre vs. Post) as the within-subjects factor and Group (MF vs. CON) as the between-subjects factor. For mental demand, a significant Group × Session interaction was observed, *F*(1, 20) = 44.4, *p* < 0.001, η^2^ₚ = 0.689, 90% CI [0.45, 0.79], indicating a large effect. This reflects a greater increase in mental demand from baseline to post-condition in the MF group compared to the CON group. Post hoc comparisons (Bonferroni-adjusted) showed that mental demand significantly increased from baseline to post-condition in the MF group (*p* < 0.001, Cohen’s d = −6.45, 95% CI [−9.70, −3.20]), while a smaller but still significant increase was observed in the CON group (*p* < 0.001, Cohen’s d = −2.30, 95% CI [−3.98, −0.63]). The main effect of Session was also significant, *F*(1, 20) = 197.8, *p* < 0.001, η^2^ₚ = 0.908, 90% CI [0.82, 0.94], representing a large effect, indicating an overall increase in mental demand across sessions. The main effect of Group was significant as well, *F*(1, 20) = 62.5, *p* < 0.001, η^2^ₚ = 0.758, 90% CI [0.55, 0.83], also reflecting a large effect, with higher mental demand reported overall in the MF group compared to the CON group.

The Group × Session interaction for physical demand was not significant, *F*(1, 20) = 1.79, *p* = 0.196, η^2^ₚ = 0.082, indicating no differential change in physical demand between the MF group and the CON group across sessions. The main effect of Session was also non-significant, *F*(1, 20) = 1.79, *p* = 0.196, η^2^ₚ = 0.082, reflecting no overall change in physical demand from baseline to post-condition. Similarly, the main effect of Group was not significant, *F*(1, 20) = 2.31, *p* = 0.144, η^2^ₚ = 0.104, suggesting no overall difference in physical demand between the MF and CON groups.

The Group × Session interaction for effort was not significant, *F*(1, 20) = 4.10, *p* = 0.056, η^2^ₚ = 0.170, indicating no differential change in effort between the MF group and the CON group across sessions. The main effect of Session was not significant, *F*(1, 20) = 4.10, *p* = 0.056, η^2^ₚ = 0.170, suggesting no overall change in effort from baseline to post-condition. Similarly, the main effect of Group was not significant, *F*(1, 20) = 0.24, *p* = 0.631, η^2^ₚ = 0.012, indicating no overall difference in effort between the MF and CON groups.

The Group × Session interaction for frustration was not significant, *F*(1, 20) = 0.16, *p* = 0.697, η^2^ₚ = 0.008, indicating no differential change in frustration between the MF group and the CON group across sessions. The main effect of Session was not significant, *F*(1, 20) = 3.91, *p* = 0.062, η^2^ₚ = 0.163, suggesting no overall change in frustration levels across sessions. The main effect of Group was not significant, *F*(1, 20) = 0.06, *p* = 0.810, η^2^ₚ = 0.003, indicating similar frustration levels between the MF and CON groups.

An independent samples *t*-test was conducted to compare mental demand ratings after the experimental condition (Stroop task) between the Mental Fatigue (MF) and Control (CON) groups. The analysis revealed a significant difference, *t*(20) = 11.98, *p* < 0.001, Cohen’s d = 5.11, 95% CI [3.31, 6.87] indicating a large effect. The MF group reported substantially higher mental demand (M = 60.9, SD = 8.31) compared to the CON group (M = 25.5, SD = 5.22). The 95% confidence interval for the mean difference ranged from 3.31 to 6.87. No significant differences were found for physical demand, *t*(20) = 1.67, *p* = 0.111, Cohen’s d = 0.71, 95% CI [−0.16, 1.57], (MF: M = 16.4, SD = 8.09; CON: M = 11.8, SD = 4.05), or frustration, *t*(20) = 1.22, *p* = 0.235, Cohen’s d = 0.52, 95% CI [−0.33, 1.37], (MF: M = 28.2, SD = 9.82; CON: M = 22.7, SD = 11.04). In contrast, effort ratings were significantly higher in the MF group compared to the CON group, *t*(20) = 12.33, *p* < 0.001, Cohen’s d = 5.26, 95% CI [3.42, 7.06], with the MF group reporting greater effort (M = 40.9, SD = 8.31) than the CON group (M = 10.0, SD = 0.00). Moreover, an independent samples *t*-test was conducted to compare subjective mental fatigue ratings (VAS) between the MF group and the CON group following the experimental condition. The analysis revealed a significant difference between groups, *t*(20) = 8.85, *p* < 0.001, Cohen’s d = 3.78, 95% CI [2.33, 5.19], representing a large effect, indicating substantially higher subjective mental fatigue in the MF group (M = 7.45, SD = 1.13) compared to the CON group (M = 3.91, SD = 0.70).

**Figure 8:**
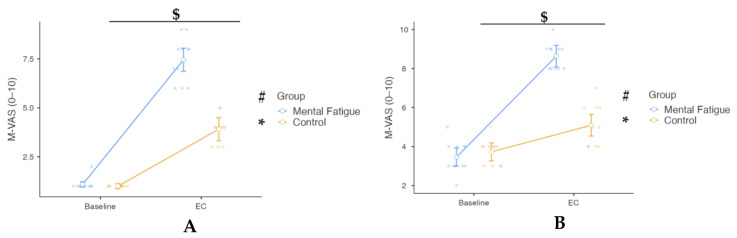
Subjective mental fatigue (M-VAS) ratings across experimental phases. Note: M-VAS ratings (mean ± SE, scale 0–10) for the Mental Fatigue (MF) and Control (CON) groups across sessions (Pre = baseline; EC = experimental condition). Symbols indicate: $ = significant Group × Session interaction; * = significant main effect of Session (time); # = significant main effect of Group (condition). MF = Mental Fatigue group; CON = Control group; EC = Experimental Condition. (**A**) M-VAS ratings from baseline compared to ratings immediately after the experimental cognitive task (Stroop test), used to induce mental fatigue (manipulation check). A significant Group × Session interaction ($ *p* < 0.05), a significant main effect of Session (* *p* < 0.05), and a significant main effect of Group (# *p* < 0.05) were observed. (**B**) M-VAS ratings from baseline (after all physical performance tests) compared to ratings immediately after the post-intervention condition (post-mental fatigue or control exposure and subsequent physical performance tests). A significant Group × Session interaction ($ *p* < 0.05), a significant main effect of Session (* *p* < 0.05), and a significant main effect of Group (# *p* < 0.05) were observed, reflecting a greater increase in subjective mental fatigue in the MF group compared to the CON group.

Subjective mental fatigue (M-VAS) ratings were analyzed using a two-way mixed-design repeated-measures ANOVA, with Session (Baseline vs. Post-task) as the within-subjects factor to evaluate the effectiveness of the cognitive fatigue manipulation, by comparing M-VAS ratings from baseline and immediately after the EC cognitive task and Group (MF vs. CON) as the between-subjects factor (Figure 8A). A significant Group × Session interaction was observed, *F*(1, 20) = 75.20, *p* < 0.001, η^2^ₚ = 0.790, 90% CI [0.61, 0.86], indicating a large effect. Post hoc Bonferroni-adjusted comparisons revealed that M-VAS ratings significantly increased from Baseline to Post-task in both groups; however, the increase was substantially greater in the MF group (*p* < 0.001, Cohen’s d = −9.35, 95% CI [−13.84, −4.85]), compared to the CON group (*p* < 0.001, Cohen’s d = −4.27, 95% CI [−6.59, −1.95]). The main effect of Session was significant, *F*(1, 20) = 541.90, *p* < 0.001, η^2^ₚ = 0.964, 90% CI [0.93, 0.98], reflecting a large overall increase in subjective mental fatigue following the cognitive task. A significant main effect of Group was also found, *F*(1, 20) = 74.10, *p* < 0.001, η^2^ₚ = 0.787, 90% CI [0.60, 0.85], with higher overall M-VAS ratings in the MF group than in the CON group. These results confirm the effectiveness of cognitive fatigue manipulation in eliciting subjective mental fatigue.

M-VAS scores were analyzed using a two-way mixed-design repeated-measures ANOVA, with Session (Baseline after all tests vs. Post-intervention after all tests) as the within-subjects factor and Group (MF vs. CON) as the between-subjects factor. A significant Group × Session interaction was observed, *F*(1, 20) = 72.30, *p* < 0.001, η^2^ₚ = 0.783, 90% CI [0.60, 0.85], indicating a large effect. Post hoc Bonferroni-adjusted comparisons revealed that M-VAS scores increased significantly from baseline to post-intervention in both groups, with a greater increase in the MF group (mean difference = 5.18, *p* < 0.001, Cohen’s d = −6.38, 95% CI [−9.55, −3.22]) than in the CON group (mean difference = 1.64, *p* = 0.002, Cohen’s d = −1.68, 95% CI [−3.06, −0.30]). The main effect of Session was also significant, *F*(1, 20) = 212.50, *p* < 0.001, η^2^ₚ = 0.914, 90% CI [0.83, 0.94], reflecting a large overall increase in subjective mental fatigue across sessions. Additionally, the main effect of Group was significant, *F*(1, 20) = 38.60, *p* < 0.001, η^2^ₚ = 0.659, 90% CI [0.40, 0.77], indicating higher overall M-VAS scores in the MF group compared to the CON group (Figure 8B).

**Figure 9:**
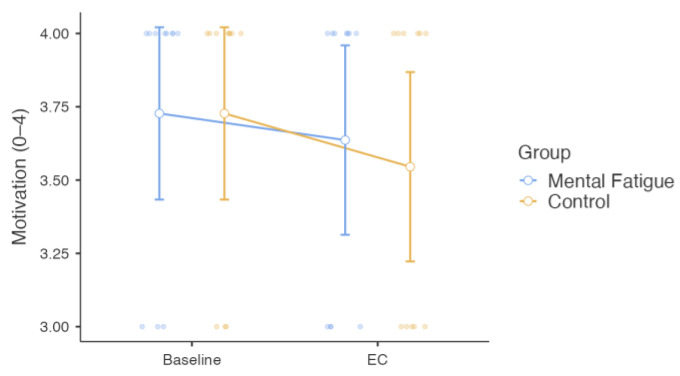
Motivation Levels. Note: Motivation ratings (mean ± SE) for the Mental Fatigue (MF) and Control (CON) groups across sessions (Pre = baseline; EC = experimental condition). No significant Group × Session interaction, main effect of Session, or main effect of Group was observed. MF = Mental Fatigue group; CON = Control group; EC = Experimental Condition.

The Group × Session interaction for motivation, analyzed using a two-way mixed-design repeated-measures ANOVA, was not significant, *F*(1, 20) = 0.36, *p* = 0.557, η^2^ₚ = 0.018, indicating no differential change in motivation between the MF group and the CON group across sessions. The main effect of Session was not significant, *F*(1, 20) = 3.21, *p* = 0.088, η^2^ₚ = 0.138, indicating no overall change in motivation across sessions. The main effect of Group was also non-significant, *F*(1, 20) = 0.05, *p* = 0.818, η^2^ₚ = 0.003, suggesting no difference in motivation levels between groups (Figure 9).

**Figure 10:**
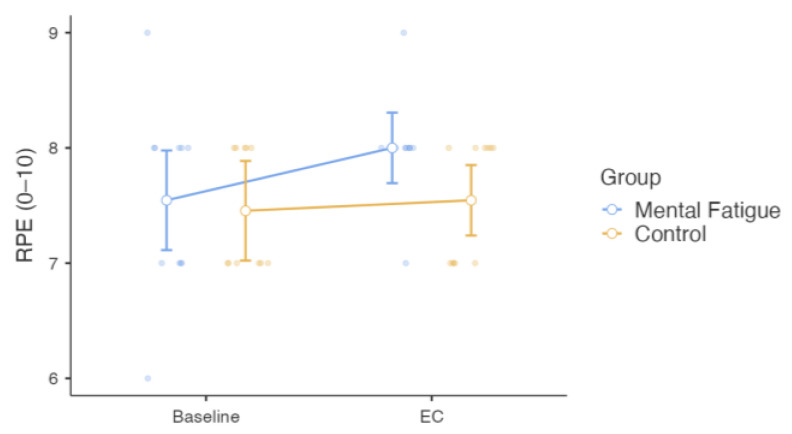
Effect of Mental Fatigue on Rating of Perceived Exertion (RPE). Note: RPE (mean ± SE, scale 0–10) for the Mental Fatigue (MF) and Control (CON) groups across sessions (Pre = baseline; EC = experimental condition). No significant Group × Session interaction, main effect of Session, or main effect of Group was observed. MF = Mental Fatigue group; CON = Control group; EC = Experimental Condition.

RPE was analyzed using a two-way mixed-design repeated-measures ANOVA, where the Group × Session interaction for RPE was not significant, *F*(1, 20) = 1.51, *p* = 0.233, η^2^ₚ = 0.070, indicating no differential change in RPE between the MF group and the CON group across sessions. The main effect of Session was not significant, *F*(1, 20) = 3.40, *p* = 0.080, η^2^ₚ = 0.145, showing no overall change in RPE across sessions. The main effect of Group was also not significant, *F*(1, 20) = 1.75, *p* = 0.201, η^2^ₚ = 0.080, indicating comparable RPE levels between groups (Figure 10).

## 4. Discussion

This study investigated the selective impact of mental fatigue (MF) on physical strength, explosive power, psychomotor performance, and subjective workload in physically active young adults. The primary finding was that MF significantly impaired non-dominant handgrip strength and reaction time, while dominant handgrip strength, back strength, and squat jump performance remained unaffected. These outcomes align with prior research demonstrating the asymmetric vulnerability of neuromuscular function under cognitive load, particularly in less automatized, cortically demanding movements involving the non-dominant limb [12,18,19]. 

The reduction in non-dominant handgrip strength supports the hypothesis that MF disproportionately impairs tasks requiring greater cortical engagement. Jacquet et al. [12] attributed similar impairments to neurophysiological changes—specifically reduced attention-related ERP components and increased alpha inhibition—which interfere with motor coordination and voluntary drive without affecting peripheral muscle activation. Additionally, asymmetries may reflect underlying cortical organization: the dominant hemisphere typically exhibits more efficient motor cortex activation, while the non-dominant side may rely on broader and more resource-intensive cortical recruitment. Under conditions of MF, attentional resources may become insufficient to support precise control of the less-practiced limb, increasing susceptibility to central performance decrements [18,19]. This finding aligns with the dynamic-dominance hypothesis proposed by Sainburg [67], which posits that dominant and non-dominant limbs utilize distinct motor control strategies—predictive control in the dominant limb and feedback-driven control in the non-dominant. Under mental fatigue, these differences may be amplified, especially in tasks requiring precision or sustained attention. Furthermore, a growing body of evidence indicates that fatigue exacerbates inter-limb asymmetries in both biomechanical and performance metrics. For instance, Gao et al. [68] demonstrated increased asymmetry in lower-limb joint mechanics following running-induced fatigue, highlighting altered symmetry angles in joint rotation and moment generation. Similarly, a systematic review by Heil et al. [69] summarized evidence across multiple sports and exercise modalities, concluding that exercise-induced fatigue can lead to asymmetries that potentially elevate injury risk during dynamic, sport-specific actions.

No significant changes were observed in isometric back strength or squat jump height, consistent with evidence that MF does not impair maximal voluntary contraction or brief, high-intensity single efforts [3,13,14]. Pageaux and Lepers [3] proposed that MF primarily disrupts prolonged or skill-based performance through elevated perceived effort, rather than through direct neuromuscular limitations. These findings likely explain why isolated efforts like squat jump and back strength remained intact in the current study.

Although the interaction effect size for squat jump height was moderate (η^2^ₚ = 0.155), it did not reach statistical significance (*p* = 0.070). Prior studies suggest that MF may impair repeated explosive efforts more than single actions [21], but the current design included only isolated trials, precluding confirmation of this effect.

The clearest effect of MF emerged in psychomotor vigilance, with a marked and statistically significant increase in reaction time in the MF group (Δ = 117.82 ms), while only a marginal, non-significant change occurred in the Control group. These results are consistent with previous findings that MF impairs sustained attention [7], compromises response planning and decision-making speed [21], and reduces vigilance stability over time [23]. Although the number of lapses did not differ significantly between groups, a descriptive increase in the MF group may indicate a cumulative impact of cognitive strain on attentional performance, though this interpretation remains speculative and should be confirmed in future studies [23].

A growing body of literature, including the works of Enoka and Duchateau [70] and Behrens et al. [71], supports a multidimensional framework for understanding fatigue, which distinguishes between performance fatigability—a measurable decline in physical or cognitive performance—and perceived fatigability—the subjective experience of fatigue that regulates task engagement and effort allocation. These two domains are conceptually distinct yet interdependent, influenced by both task demands and individual characteristics such as attentional capacity, motivation, and affective state. In the present study, objective outcomes (e.g., maximal voluntary contraction and Stroop task performance) aligned primarily with performance fatigability, whereas M-VAS and NASA-TLX scores captured perceived fatigability. The dissociation observed between these domains reinforces the notion that mental fatigue may selectively impair perception-driven components of performance without uniformly degrading motor output. This theoretical distinction was reflected in the present study’s subjective workload data, which confirmed the successful induction of MF. Significant increases in NASA-TLX Mental Demand and M-VAS scores in the MF group replicated prior findings that MF elevates perceived effort without necessarily altering physiological output [3,10,21]. These results reflect the psychobiological model of fatigue, which emphasizes central perceptual changes—such as altered effort-cost evaluation—as primary mediators of task disengagement and performance reduction [3,11,12]. Notably, effort ratings (NASA-TLX) increased significantly post-intervention, while RPE showed a numerical increase that did not reach statistical significance (*p* = 0.080), particularly in the MF group. This partial dissociation supports prior findings that MF elevates subjective exertion in endurance contexts even when physical outputs remain stable [3,10]. In this study, the lower-intensity and shorter duration of physical tasks may have attenuated perceptual divergence across conditions.

M-VAS ratings of subjective fatigue also increased significantly and were sustained throughout the post-intervention phase, consistent with research showing that perceived cognitive fatigue persists beyond the task itself and may affect subsequent motor and cognitive performance [21,22,23]. These enduring effects highlight the cumulative nature of MF and its potential to disrupt task regulation and engagement over time [11,22].

Taken together, the results indicate that while MF does not consistently impair gross motor strength or single maximal outputs, it meaningfully alters subjective experience and selectively impairs fine motor control and vigilance, especially in the non-dominant limb. These outcomes reinforce the central role of perceptual and cognitive mechanisms in mediating performance under mental fatigue [3,10,12]. This distinction between central and peripheral fatigue underscores the vulnerability of non-automatized movements and attention-dependent tasks to cognitive strain, while more automated, gross motor tasks remain comparatively unaffected [3,10,11,12,13,72].

From an applied perspective, these findings suggest that cognitive fatigue can undermine performance in tasks requiring fine control or rapid response, even in the absence of gross motor impairment. Practitioners should consider the timing of cognitively demanding activities—such as video analysis, tactical briefings, or academic commitments—relative to training and competition. Tools like the M-VAS and NASA-TLX, as well as wearable or app-based cognitive monitoring, may offer valuable insights into athletes’ readiness and guide load management strategies.

Furthermore, Brain Endurance Training (BET) has emerged as a promising approach for enhancing cognitive resilience and mitigating the effects of MF [24,29,30,31]. When guided by evidence-based protocols and tailored to sport-specific demands, BET may improve athletes’ tolerance to cognitive stress. Additionally, reducing unregulated digital exposure (e.g., excessive screen time) may help preserve attentional resources and optimize performance readiness.

Despite the strengths of this study, several limitations should be noted. Inter-individual variability in fatigue sensitivity, baseline cognitive function, and fitness levels may have influenced outcomes despite randomization. Although the sample was gender-balanced, the limited number of participants precluded subgroup analyses by sex, training background, or fatigue responsiveness. Future studies with larger samples should explore whether these factors moderate MF susceptibility. Furthermore, the use of a between-subjects design helped avoid carryover effects but may have reduced statistical sensitivity. A within-subject crossover design could enhance detection of more subtle MF effects. Lastly, the absence of physiological markers (e.g., EEG, HRV, cortisol) limits interpretation of underlying mechanisms. Including such measures would provide a more complete understanding of the neurocognitive basis of MF.

## 5. Conclusions

This study shows that acute mental fatigue, induced via a Stroop task, selectively impairs non-dominant handgrip strength and psychomotor vigilance in physically active young adults. In contrast, dominant handgrip, isometric back strength, and squat jump height remained unaffected, suggesting that well-automatized, gross motor tasks are more resilient to transient cognitive load. The asymmetrical decline in non-dominant strength likely reflects higher cortical demands for motor control, while the marked slowing in reaction time confirms degraded attentional efficiency. Though lapses did not reach significance, the substantial increase in response latency reinforces MF’s impact on cognitive processing. These findings highlight the importance of managing cognitive load in settings where fine motor control and sustained attention are critical. Future research should explore MF’s effects on repeated-effort tasks and evaluate interventions—such as dual-task or brain endurance training—that may enhance resilience under cognitive strain.

## Data Availability

The data presented in this study are available upon reasonable request from the corresponding author because of restrictions.

## References

[1] Boksem M.A., Tops M. (2008). Mental fatigue: Costs and benefits. Brain Res. Rev..

[2] Boksem M.A., Meijman T.F., Lorist M.M. (2006). Mental fatigue, motivation and action monitoring. Biol. Psychol..

[3] Pageaux B., Lepers R. (2018). The effects of mental fatigue on sport-related performance. Prog. Brain Res..

[4] Russell S., Jenkins D., Smith M., Halson S., Kelly V. (2019). The application of mental fatigue research to elite team sport performance: New perspectives. J. Sci. Med. Sport.

[5] Russell S., Jenkins D., Rynne S., Halson S.L., Kelly V. (2019). What is mental fatigue in elite sport? Perceptions from athletes and staff. Eur. J. Sport Sci..

[6] Yuan R., Sun H., Soh K.G., Mohammadi A., Toumi Z., Zhang Z. (2023). The effects of mental fatigue on sport-specific motor performance among team sport athletes: A systematic scoping review. Front. Psychol..

[7] Habay J., Van Cutsem J., Verschueren J., De Bock S., Proost M., De Wachter J., Roelands B. (2021). Mental fatigue and sport-specific psychomotor performance: A systematic review. Sports Med..

[8] Smith M.R., Thompson C., Marcora S.M., Skorski S., Meyer T., Coutts A.J. (2018). Mental fatigue and soccer: Current knowledge and future directions. Sports Med..

[9] Smith M.R., Marcora S.M., Coutts A.J. (2015). Mental fatigue impairs intermittent running performance. Med. Sci. Sports Exerc..

[10] Staiano W., Bonet L.R.S., Romagnoli M., Ring C. (2023). Mental fatigue: The cost of cognitive loading on weight lifting, resistance training, and cycling performance. Int. J. Sports Physiol. Perform..

[11] Li T., Zhang D., Wang Y., Cheng S., Wang J., Zhang Y., Chen X. (2024). Research on mental fatigue during long-term motor imagery: A pilot study. Sci. Rep..

[12] Jacquet T., Poulin-Charronnat B., Bard P., Lepers R. (2024). Effect of mental fatigue on hand force production capacities. PLoS ONE.

[13] Alix-Fages C., Baz-Valle E., González-Cano H., Jiménez-Martínez P., Balsalobre-Fernández C. (2023). Mental fatigue from smartphone use or Stroop task does not affect bench press force–velocity profile, one-repetition maximum, or vertical jump performance. Mot. Control.

[14] Holgado D., Jolidon L., Borragán G., Sanabria D., Place N. (2023). Individualized mental fatigue does not impact neuromuscular function and exercise performance. Med. Sci. Sports Exerc..

[15] Ramsay E., Alizadeh S., Summers D., Hodder A., Behm D.G. (2023). The effect of a mental task versus unilateral physical fatigue on non-local muscle fatigue in recreationally active young adults. J. Sports Sci. Med..

[16] Alix-Fages C., Grgic J., Jiménez-Martínez P., Baz-Valle E., Balsalobre-Fernández C. (2022). Effects of mental fatigue on strength endurance: A systematic review and meta-analysis. Mot. Control.

[17] Fortes L.S., Gantois P., de Lima-Júnior D., Barbosa B.T., Ferreira M.E.C., Nakamura F.Y., Fonseca F.S. (2023). Playing videogames or using social media applications on smartphones causes mental fatigue and impairs decision-making performance in amateur boxers. Appl. Neuropsychol. Adult.

[18] Türkmen O.B., Akçay B., Demir C., Kurtoğlu A., Alotaibi M.H., Elkholi S.M. (2024). Does the effect of mental fatigue created by motor imagery on upper extremity functions change with diaphragmatic breathing exercises? A randomized, controlled, single-blinded trial. Medicina.

[19] Severijns D., Lamers I., Kerkhofs L., Feys P. (2015). Hand grip fatigability in persons with multiple sclerosis according to hand dominance and disease progression. J. Rehabil. Med..

[20] Jacquet T., Lepers R., Poulin-Charronnat B., Bard P., Pfister P., Pageaux B. (2021). Mental fatigue induced by prolonged motor imagery increases perception of effort and the activity of motor areas. Neuropsychologia.

[21] Staiano W., Bonet L.R.S., Romagnoli M., Ring C. (2024). Mental fatigue impairs repeated sprint and jump performance in team sport athletes. J. Sci. Med. Sport.

[22] Díaz-García J., Clemente-Suárez V.J., Fuentes-García J.P., Villafaina S. (2023). Combining HIIT plus cognitive task increased mental fatigue but not physical workload in tennis players. Appl. Sci..

[23] Angius L., Merlini M., Hopker J., Bianchi M., Fois F., Piras F., Cugia P., Russell J., Marcora S.M. (2022). Physical and mental fatigue reduce psychomotor vigilance in professional football players. Int. J. Sports Physiol. Perform..

[24] Dallaway N., Mortimer H., Gore A., Ring C. (2024). Brain endurance training improves dynamic calisthenic exercise and benefits novel exercise and cognitive performance: Evidence of performance enhancement and near transfer of training. J. Strength Cond. Res..

[25] Daub B.D., McLean B.D., Heishman A.D., Peak K.M., Coutts A.J. (2022). The relationship between mental fatigue and shooting performance over the course of a National Collegiate Athletic Association Division I basketball season. J. Strength Cond. Res..

[26] Daub B.D., McLean B.D., Heishman A.D., Peak K.M., Coutts A.J. (2023). Impacts of mental fatigue and sport-specific film sessions on basketball shooting tasks. Eur. J. Sport Sci..

[27] López-Rodriguez R., Ring C., Díaz-García J. (2025). The detrimental effects of mental fatigue on cognitive and physical performance in older adults are accentuated by age and attenuated by habitual physical activity. J. Aging Phys. Act..

[28] Mortimer H., Dallaway N., Ring C. (2024). Effects of isolated and combined mental and physical fatigue on motor skill and endurance exercise performance. Psychol. Sport Exerc..

[29] Staiano W., Díaz-García J., García-Calvo T., Ring C. (2025). Brain endurance training improves soccer-specific technical skills and cognitive performance in fatigued professional soccer players. J. Sci. Med. Sport.

[30] Staiano W., Marcora S., Romagnoli M., Kirk U., Ring C. (2023). Brain Endurance Training Improves Endurance and Cognitive tPerformance in Road Cyclists. J. Sci. Med. Sport.

[31] Staiano W., Merlini M., Romagnoli M., Kirk U., Ring C., Marcora S. (2022). Brain endurance training improves physical, cognitive, and multitasking performance in professional football players. Int. J. Sports Physiol. Perform..

[32] Staiano W., Romagnoli M., Salazar Bonet L.R., Ferri-Caruana A. (2024). Adaptive cognitive tasks for mental fatigue: An innovative paradigm for cognitive loading in human performance. J. Sci. Med. Sport.

[33] Waldman H.S., O’Neal E.K., Barker G.A., Witt C.R., Lara D.A., Huber A.K., Egan B. (2023). No benefit of ingesting a low-dose ketone monoester supplement on markers of cognitive performance in females. J. Cogn. Enhanc..

[34] Waldman H.S., O’Neal E.K., Barker G.A., Witt C.R., Lara D.A., Huber A.K., Egan B. (2024). A ketone monoester with carbohydrate improves cognitive measures postexercise, but not performance in trained females. Med. Sci. Sports Exerc..

[35] Stroop J.R. (1935). Studies of interference in serial verbal reactions. J. Exp. Psychol..

[36] Migliaccio G.M., Di Filippo G., Russo L., Orgiana T., Ardigò L.P., Casal M.Z., Padulo J. (2022). Effects of mental fatigue on reaction time in sportsmen. Int. J. Environ. Res. Public Health.

[37] Penna E.M., Filho E., Wanner S.P., Campos B.T., Quinan G.R., Mendes T.T., Smith M.R., Prado L.S. (2018). Mental fatigue impairs physical performance in young swimmers. Pediatr. Exerc. Sci..

[38] Rauch W.A., Schmitt K. Fatigue of cognitive control in the Stroop-task. Proceedings of the 31st Annual Conference of the Cognitive Science Society.

[39] Scarpina F., Tagini S. (2017). The Stroop color and word test. Front. Psychol..

[40] Dinges D.F., Powell J.W. (1985). Microcomputer analyses of performance on a portable, simple visual RT task during sustained operations. Behav. Res. Methods Instrum. Comput..

[41] Rubio-Morales A., Díaz-García J., Barbosa C., Habay J., López-Gajardo M.Á., García-Calvo T. (2022). Do cognitive, physical, and combined tasks induce similar levels of mental fatigue? Testing the effects of different moderating variables. Mot. Control.

[42] Proost M., Habay J., De Wachter J., De Pauw K., Rattray B., Meeusen R., Van Cutsem J. (2022). How to tackle mental fatigue: A systematic review of potential countermeasures and their underlying mechanisms. Sports Med..

[43] Hart S.G., Staveland L.E. (1988). Development of NASA-TLX (Task Load Index): Results of empirical and theoretical research. Advances in Psychology.

[44] Grier R.A. (2015). How high is high? A meta-analysis of NASA-TLX global workload scores. Proceedings of the Human Factors and Ergonomics Society Annual Meeting.

[45] Díaz-García J., González-Ponce I., Ponce-Bordón J.C., López-Gajardo M.Á., Ramírez-Bravo I., Rubio-Morales A., García-Calvo T. (2021). Mental load and fatigue assessment instruments: A systematic review. Int. J. Environ. Res. Public Health.

[46] Borg G. (1982). Ratings of perceived exertion and heart rates during short-term cycle exercise and their use in a new cycling strength test. Int. J. Sports Med..

[47] Balsalobre-Fernández C., Varela-Olalla D. (2024). The validity and reliability of the My Jump Lab App for the measurement of vertical jump performance using artificial intelligence. Sensors.

[48] Balsalobre-Fernández C., Glaister M., Lockey R.A. (2015). The validity and reliability of an iPhone app for measuring vertical jump performance. J. Sports Sci..

[49] Bogataj Š., Pajek M., Andrašić S., Trajković N. (2020). Concurrent validity and reliability of My Jump 2 app for measuring vertical jump height in recreationally active adults. Appl. Sci..

[50] Puljić D., Karavas C., Mandroukas A., Stafylidis A. (2024). Validity of the Enode Sensor and My Jump 3 App for assessing countermovement jump performance. Appl. Sci..

[51] Ding L., Lyu M., Chen Z., Wu J., Wang Y., Bishop C., Li Y. (2024). Associations between inter-limb asymmetry in lower limb strength and jump performance in 14–15-year-old basketball players. Symmetry.

[52] Wang Y.C., Bohannon R.W., Li X., Yen S.C., Sindhu B., Kapellusch J. (2019). Summary of grip strength measurements obtained in the 2011–2012 and 2013–2014 National Health and Nutrition Examination Surveys. J. Hand Ther..

[53] Balogun J.A., Onigbinde A.T. (1991). Intratester reliability and validity of the Takei Kiki Kogo hand grip dynamometer. J. Phys. Ther. Sci..

[54] Watanabe T., Owashi K., Kanauchi Y., Mura N., Takahara M., Ogino T. (2005). The short-term reliability of grip strength measurement and the effects of posture and grip span. J. Hand Surg..

[55] Coldwells A., Atkinson G., Reilly T. (1994). Sources of variation in back and leg dynamometry. Ergonomics.

[56] Dobbin N., Hunwicks R., Jones B., Till K., Highton J., Twist C. (2018). Criterion and construct validity of an isometric midthigh-pull dynamometer for assessing whole-body strength in professional rugby league players. Int. J. Sports Physiol. Perform..

[57] Imagama S., Matsuyama Y., Hasegawa Y., Sakai Y., Ito Z., Ishiguro N., Hamajima N. (2011). Back muscle strength and spinal mobility are predictors of quality of life in middle-aged and elderly males. Eur. Spine J..

[58] Kroll P.G., Machado L., Happy C., Leong S., Chen B. (2000). The relationship between five measures of trunk strength. J. Back Musculoskelet. Rehabil..

[59] Díaz-García J., García-Calvo T., López-Gajardo M.A., Rubio-Morales A., Parraca J.A. (2023). Physical fatigue exacerbates the negative effects of mental fatigue on soccer performance in practitioners. Eur. J. Hum. Mov..

[60] Queirós V.S.D., Dantas M., Fortes L.D.S., Silva L.F.D., Silva G.M.D., Dantas P.M.S., Cabral B.G.D.A.T. (2021). Mental fatigue reduces training volume in resistance exercise: A cross-over and randomized study. Percept. Mot. Ski..

[61] Cohen J. (1988). Statistical Power Analysis for the Behavioral Sciences.

[62] Richardson J.T.E. (2011). Eta squared and partial eta squared as measures of effect size in educational research. Educ. Res. Rev..

[63] Lakens D. (2016). Improving Your Statistical Inferences. https://lakens.github.io/statistical_inferences/.

[64] Wuensch K.L. (2017). SPSS Programs (Syntax).

[65] Steiger J.H. (2004). Beyond the F Test: Effect Size Confidence Intervals and Tests of Close Fit in the Analysis of Variance and Contrast Analysis. Psychol. Methods.

[66] Smithson M. (2003). Applications in ANOVA and Regression. Confidence Intervals.

[67] Sainburg R. (2002). Evidence for a Dynamic-Dominance Hypothesis of Handedness. Exp. Brain Res..

[68] Gao Z., Fekete G., Baker J.S., Liang M., Xuan R., Gu Y. (2022). Effects of Running Fatigue on Lower Extremity Symmetry among Amateur Runners: From a Biomechanical Perspective. Front. Physiol..

[69] Heil J., Loffing F., Büsch D. (2020). The Influence of Exercise-Induced Fatigue on Inter-Limb Asymmetries: A Systematic Review. Sports Med. Open.

[70] Enoka R.M., Duchateau J. (2016). Translating Fatigue to Human Performance. Med. Sci. Sports Exerc..

[71] Behrens M., Gube M., Chaabene H., Prieske O., Zenon A., Broscheid K.-C., Schega L., Husmann F., Weippert M. (2023). Fatigue and Human Performance: An Updated Framework. Sports Med..

[72] Marcora S.M., Staiano W., Manning V. (2009). Mental fatigue impairs physical performance in humans. J. Appl. Physiol..

